# Pediatric Respiratory Viral Infection

**DOI:** 10.3390/v16050733

**Published:** 2024-05-06

**Authors:** Stacy L. S. Yam, Joan Marie Javillo Baguio, Renee W. Y. Chan

**Affiliations:** 1 Department of Paediatrics, Faculty of Medicine, The Chinese University of Hong Kong (CUHK), Hong Kong, China; 2 Hong Kong Hub of Paediatric Excellence, The Chinese University of Hong Kong (CUHK), Hong Kong, China; 3 Laboratory for Paediatric Respiratory Research, Li Ka Shing Institute of Health Sciences, The Chinese University of Hong Kong (CUHK), Hong Kong, China; 4 SH Ho Research Centre for Infectious Diseases Faculty of Medicine, The Chinese University of Hong Kong (CUHK), Hong Kong, China

Reflecting on this Special Issue dedicated to pediatric respiratory viruses, it is evident that the shadow cast by the global SARS-CoV-2 pandemic has profoundly impacted individuals of all ages and backgrounds, neonates and school-aged children being vulnerable cohorts resulting from the evolving immunological profiles and limited exposures to immunity-building experienced during this unprecedented era. Recognizing the distinctive interplay between COVID-19 and pediatric patients, from the initial clinical presentation to treatment modalities and enduring post-infection consequences, becomes imperative. Thus, a nuanced and tailored approach to pediatric care is warranted, as children transcend the mere replication of adults.

The surge of COVID-19 cases placed an unprecedented strain on healthcare systems worldwide, profoundly impacting hospitalization rates and management strategies. Notably, research conducted in Germany unveiled the significant financial toll associated with respiratory viruses, offering invaluable insights into the financial burdens borne by healthcare facilities, both in ICU and non-ICU settings, even prior to the COVID-19 era.

Within the innovation realm, the contributions of Dobrijevic et al. heralded the advent of rapid triage protocols, emphasizing the utmost significance of precise severity assessments. These protocols assume a pivotal role in expediting patient care, particularly in situations where immediate qPCR results are unattainable; such initiatives not only optimize resource allocation but also underscore the urgent need to evaluate the pandemic’s implications in monetary terms.

Moreover, amidst concerns regarding the exacerbation of pre-existing respiratory and immunological conditions by COVID-19, studies from Japan and Taiwan have provided nuanced perspectives. While reassuring findings from Wakiguchi’s team suggest no severe cases or deaths among children with rheumatic diseases and COVID-19, Sung’s research highlights the heightened risk of acute bronchitis and bronchiolitis in children with asthma in the pre-COVID-19 era, which may pose the additional risk of exacerbation upon COVID-19 infection that requires further consideration. Such nuanced perspectives are pivotal in informing treatment options and maximizing outcomes for pediatric patients grappling with comorbidities. Furthermore, the intersectionality of COVID-19 with other respiratory viruses, including Respiratory Syncytial Virus (RSV) and rhinoviruses, underscores the complexity of pediatric respiratory health. The occurrence of co-infections involving COVID-19 has been demonstrated to worsen outcomes, particularly among neonates, with environmental factors, seasonality, and temperature, as an example, emerging as influential factors in disease transmission dynamics and mitigation strategies.

To round up this Special Issue, with a particular emphasis on the impact of COVID-19, this comprehensive exploration of pediatric respiratory viral infections resounds as an urgent clarion call for enhanced management strategies founded on the profound comprehension of disease burden, diagnostic advancements, and treatment modalities. By embracing the intricate nature of developing immunity, childhood disorders, and the broader societal and environmental dynamics at play, we can forge a path towards more resilient and responsive pediatric healthcare systems, ensuring the well-being of our youngest and most vulnerable patients.

